# Sustained Delivery of Rifampicin Nanoformulation Administration Intravitreally Into Rabbit Eyes for Ocular Tuberculosis

**DOI:** 10.7759/cureus.65368

**Published:** 2024-07-25

**Authors:** Rohitash Yadav, Chakrmani Tiwari, Vinod Kumar, Avaneesh Pandey, Ritika Kondel, Nushrat Shafiq

**Affiliations:** 1 Pharmacology, Postgraduate Institute of Medical Education and Research, Chandigarh, IND; 2 Biochemistry, King George's Medical University, Lucknow, IND; 3 Pharmacology, All India Institute of Medical Sciences, Rishikesh, IND

**Keywords:** plga nanoparticles, rifampicin, ocular tuberculosis, intravitreous drug administration, anti-tuberculosis therapy

## Abstract

Introduction: Diagnosis and treatment of ocular tuberculosis is very challenging. It poses a significant and potential management dilemma after diagnosis as a primary, active, or secondary infection. The higher amounts of orally administered antitubercular drugs are needed to achieve the therapeutic concentration in the eye, which may lead to a higher risk of side effects. However, the intravitreal administration of drugs is not practiced because of the frequent administration of the injections.

Methods: This study was carried out to develop, optimize, and characterize rifampicin-loaded poly (lactic-co-glycolic acid) (PLGA) nanoparticles to make them sustained release followed by the direct administration of plain rifampicin and rifampicin nano-formulations in the vitreous of rabbit eyes. Both groups were comparatively assessed for the sustained delivery of the two preparations in the vitreous and their systemic toxicity.

Results: The characteristics of rifampicin-loaded nanoparticles were 786 nm in size with narrow size distribution along with a zeta potential of -12 mV. The drug encapsulation efficiency and loading capacity were 67.68% w/w and 42.28% w/w, respectively. The four New Zealand white rabbits were divided into two groups and given plain rifampicin (50µl volume) and PLGA nanoformulations of rifampicin (50µl volume) in each eye. In vivo, rifampicin-loaded PLGA nanoparticles produced sustained release of rifampicin for a week, even obtaining the 0.51 µg/ml levels on the seventh day in vitreous against negligible levels after one day for free rifampicin. The Cmax levels for free Rifampicin and Rifampicin nanoparticles were 0.44 µg/ml and 1.86 µg/ml, respectively.

Conclusion: In this experimental proof-of-concept study, we have found that rifampicin-loaded PLGA nanoparticles released rifampicin in a sustained manner for up to seven days compared to free drugs only for one day into the vitreous. The intravitreal-administered drug did not reach systemic circulation.

## Introduction

Ocular tuberculosis is being recognized as an important health problem. It has variable manifestations such as anterior granulomatous uveitis, anterior and posterior synechiae, secondary glaucoma, cataracts, and posterior segment manifestations, including vitritis, retinal vasculitis, and optic neuritis [1]. The currently recommended treatment of ocular tuberculosis is a four-drug regimen (Isoniazid, Rifampicin, Ethambutol, and Pyrazinamide) as for any other extraocular form of TB [2]. Compared with drug delivery to other parts of the body, ocular drug delivery has significant challenges due to various barriers [3]. When the drug is administered topically, only 5% of the drug reaches vitreous fluid due to precorneal factors and anatomical barriers [4]. Precorneal factors include solution drainage, blinking, tear ﬁlm, tear turnover, and induced lacrimation. In the case of systemic drug delivery, less than 2% reaches vitreous fluid due to the blood-aqueous barrier and blood-retinal barrier. So, a higher amount of orally administered drug is needed to achieve the therapeutic concentration in the eye, which may lead to a higher risk of side effects. These include rash, fever, nausea and vomiting, hepatotoxicity, and rarely acute renal failure. Certain types of nanoformulations can produce sustained release of drugs for many days, both in vivo and in vitro, thereby reducing the dosing frequency [5,6]. Reduction in dosing frequency may help in better management of the disease and improve patient compliance. Thus, sustained release formulations may provide more widely spaced intermittent dosing and also help to guard against the acquisition of drug resistance as there may be less chance for missed doses leading to suboptimal drug concentration at the site of action. Therefore, attempts have been made to develop intermittent therapy by applying modified-release anti-tuberculosis drug delivery systems.

Several drug delivery systems have been reported for first-line anti-tuberculosis drugs. It includes first-line antituberculosis drugs loaded PLGA nanoparticles and microparticles, alginate-based nanoparticles and microparticles, solid lipid nanoparticles (SLNs), and drug-loaded liposomes [7-11]. Among all drug delivery systems of first-line antitubercular drugs, PLGA-based nanoparticles are probably the most versatile drug delivery system due to the biodegradability, biocompatibility, and feasibility of all routes of administration [12]. PLGA nanoparticles exhibited sustained release of drugs in target organs for up to nine days, thus demonstrating the same to be the efficient carrier that can be used for the delivery of antitubercular drugs (ATDs). The work on the rifampicin was initiated on similar lines for ocular tuberculosis.

Rifampicin is an efficacious first-line drug that is widely used in the treatment of tuberculosis. It is available as conventional-release tablets that require daily administration at doses of 600 mg daily orally. PLGA-based nanoparticles can produce sustained release of rifampicin in blood for longer periods [13]. Furthermore, drug-loaded nanoparticles may improve drug efficacy by keeping the drug concentration above the minimum inhibitory concentration (MIC) for a longer period and inhibiting the growth of microorganisms. For the management of ocular tuberculosis, patients are administered first-line ATT routinely along with dexamethasone. However, there are no data available for intravitreal levels reached, and there is no evidence to suggest whether systemic administration will lead to the attainment of adequate concentration in the vitreous. To inquire into this concept, we evaluated in this proof-of-concept study whether sustained levels of rifampicin in the vitreous chamber can be attained after administration of rifampicin intravitreally into rabbit eyes.

## Materials and methods

The study was conducted after obtaining approval from the Animal Ethics Committee. The present study comprises the pilot study within the protocol. Rifampicin was purchased from GOLD BIOCOM, and Poly DL-lactide-co-glycolide was purchased from Birmingham Polymer, Inc. (Birmingham, AL). PVA (MW 30000-70000 Da, 88% hydrolyzed) was purchased from Sigma Chemical Co. (St Louis, MO, USA), and acetonitrile (ACN; HPLC grade) and dichloromethane (DCM) were obtained from Rankem Fine Chemicals (New Delhi, India) and Merck Ltd. (Mumbai, India), respectively. Ketamine HCL injection was taken from the PGIMER supply, and Xylazine injection (Indian Immunological Limited) was purchased by the Local Medical Shop, Chandigarh.

The particle size and zeta potential were analyzed by using the Zetasizer Nano ZS (Malvern Instruments, Malvern, UK). The surface morphology of the nanoparticles was analyzed by using the scanning electron microscope SU3800 (HITACHI Ltd.). Fourier Transmission Infrared Spectroscopy (FTIR) was conducted on samples of free rifampicin using a Perkin Elmer spectrometer (Perkin Elmer, Boston, MA).

Preparation of rifampicin-loaded PLGA nanoparticles

Rifampicin-loaded nanoparticles were prepared by the single emulsion solvent evaporation method as previously standardized in our lab [14-16]. Briefly, 40 mg of PLGA and 20 mg of rifampicin were dissolved in 1 ml of dichloromethane. This solution was added dropwise to an aqueous 10 ml PVA (2%, w/v) solution under homogenization for 3 minutes. The solvent was evaporated at room temperature (25°C) for 12 hours under magnetic stirring. Rifampicin-loaded nanoparticles were isolated by centrifugation at 40,000 rpm for 30 minutes (Optima XPN, Bechman Coulters, France). After centrifugation, the supernatant was recovered and assayed for unentrapped drug, and the sediment was washed using the same amount of distilled water as the supernatant and again centrifuged at 30,000 rpm for 20 minutes. The washing process was repeated thrice. All the washings were collected and assayed for free and entrapped drugs.

Animals

This research work was approved by the Institutional Animal Ethics Committee (82/IAEC/3719(63rd)), PGIMER, Chandigarh. New Zealand white rabbits were procured from the central animal house facility of the Postgraduate Institute of Medical Education and Research (PGIMER), Chandigarh, India. The animals were maintained under standard conditions with a temperature of 22 ± 2°C and a 12-hour light/dark cycle. Animals were free to access food and water. A total of four rabbits were randomly divided into only rifampicin-treated as well as rifampicin nanoformulation-treated groups (two rabbits in each group). Only drug-treated animals received 50µl volume of rifampicin (46.45 µg/ml), and the nanoformulation-treated group received 50µl volume of rifampicin nanoformulation in each eye.

Intravitreous injection in rabbit eye

**Figure 1:**
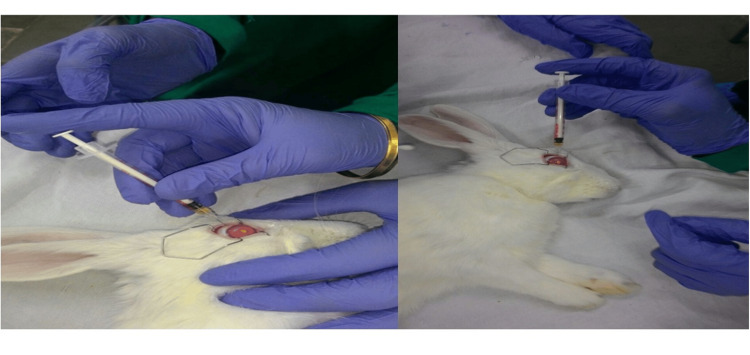
Intravitreally administration of free rifampicin and rifampicin nanoformulation into rabbit eyes A: Intravitreally administration of free rifampicin, whereas B: Intravitreally administration of rifampicin nanoformulation into rabbit eye.

The rabbits were weighed (1.5 to 2 kg) and anesthetized by the combination of 40 mg/kg ketamine hydrochloride (50mg/ml) and 12mg/kg xylazine hydrochloride administered intramuscularly. Then topical paracaine was instilled in the eyes for local anesthesia, followed by intravitreous injection (Figure 1). The eyes were kept open using an eye speculum, and saline drops were administered intermittently to prevent dryness. After anesthetization, a 50µl volume of drug was administered intravitreally 1 mm behind the surgical limbus in the superior temporal quadrant by injection with a 30-gauge needle into the vitreous cavity in each eye (Figure 1). Twenty-four hours after treatment, vitreous and blood samples were collected from the right eye of the first rabbit from each group. After treatment, vitreous samples were taken from both groups at 24 hours, 84 hours, 168 hours, and 216 hours from each eye. Twenty-four hours after treatment, a blood sample was taken from each rabbit of both groups. 

Preparation of mobile phase and standard solution of rifampicin

The mobile phase consisted of 38:62 ratios of 0.05 KH2PO4: Acetonitrile (ACN). 0.05M KH2PO4 solution was prepared by adding 3.4 gm KH2PO4 in 500 ml distilled water, after which 380 ml KH2PO4 solution was added in 620 ml acetonitrile. The pH was adjusted to 3.7 by the addition of orthophosphoric acid. A stock standard solution of rifampicin was prepared by dissolving 10 mg of the drug in 10 ml methanol (mg/ml). From the working standard solution of rifampicin, serial dilutions were prepared in the mobile phase. One ml of drug solution was added to the 9 ml mobile phase to make the concentration of 100 µg/ml. Further dilutions of various concentrations were prepared, and for each dilution, 10, 20, 40, 80, 160, and 320 µl solution were taken from 100 µg/ml concentrated drug solution and added in 990, 980, 960, 920, 840, and 680 µl mobile phase, respectively. After that, 1 ml solution of each concentration was taken in Eppendorf’s tube of 1.5 ml capacity and mixed for 2 minutes by a vortex mixture.

HPLC analyses

HPLC (high-performance liquid chromatography) analyses were performed on HPLC (Shimadzu, SPD 2A LC-20AD, Shimadzu Pump LC-20AD, Prominence liquid chromatography with SPD-20A prominence UV/Vis detector, Shimadzu Corporation, Japan). Separation of elutes was achieved with an ultra base-C18 reversed-phase column (250mm× 4.6mm i.d., 5µm particle size) (Merck Ltd. Mumbai, India). The mobile phase consisted of 0.05 M KH2PO4 and ACN (380:620v/v, pH 3.7), a flow rate of 1.5 ml/min, a temperature of 25°C, and a wavelength of 335 nm (Table 1). 

**Table 1:** HPLC conditions for estimation of rifampicin in mobile phase, vitreous, and plasma HPLC: High performance liquid chromotagraphy

HPLC Conditions	
Column	C18 Column (250×4.6 mm,5µm)
Mobile Phase	0.05M KH_2_PO_4 _(pH-3.7): Acetonitrile: 38:62
Flow rate	1.5 ml/min
Wavelength	335 nm
Calibration Curve Range	1 µg/ml – 32 µg/ml
Temp.	25^0^c

Preparation of a calibration curve of rifampicin in plasma and vitreous fluid

The rifampicin spike was determined in human plasma before HPLC estimation. Proteins in human plasma were precipitated using an equal amount of ACN and centrifuging at 1000 rpm for 5 minutes. Then, the supernatant was injected into the HPLC system to determine the peak of rifampicin. The calibration curve range was 1 to 32 µg/ml. The rifampicin peak in the vitreous was determined before the HPLC estimation of samples. Proteins in the vitreous fluid were precipitated using an equal amount of ACN and centrifuging at 1000 rpm for 5 minutes. Then, the supernatant was injected into the HPLC system to determine the peak of rifampicin. A standard curve was thus obtained.

## Results

Development of rifampicin-loaded PLGA nanoparticles

Rifampicin was successfully entrapped into PLGA nanoparticles. The nanoformulation of rifampicin was affected by processing parameters, including the drug: polymer ratio, PVA%, homogenization time, and centrifugation time. The encapsulation efficiency of 67.35% was obtained with a drug-polymer ratio of 1:2, PVA 2%, homogenization for 3 minutes, and centrifugation at 40,000 rpm.

Effect of drug-polymer ratio, PVA concentrations, and homogenization time on encapsulation efficiency

Rifampicin encapsulation efficiency was increased with increasing drug-polymer ratios (1:1 to 1:2) and attained the highest value of encapsulation efficiency with 1:2 ratios. Further increase in drug-polymer ratio (1:3 & 1:4) resulted in decreased drug encapsulation efficiency (Table 2). Rifampicin encapsulation efficiency was increased with increasing concentrations of stabilizer polyvinyl alcohol (PVA) (1-4% w/v) and attained the highest value of encapsulation efficiency with 2% w/v of PVA. Further increase in PVA concentration (3-4% w/v) resulted in decreased drug encapsulation efficiency (Table 2). Encapsulation efficiency was increased with increasing homogenization time (1-3 minutes) and attained the highest value of encapsulation efficiency with 3 minutes. Further increase time (4 minutes) resulted in decreased drug encapsulation efficiency (Table 2).

**Table 2:** Optimization of nanoparticles development by altering the drug: polymer ratio, PVA%, and homogenization time Formulations Fa, Fb, Fc, and Fd showed the effect of drug-polymer ratio on encapsulation efficacy; Formulations A, B, C, and D showed the effect of PVA% on encapsulation efficacy; whereas Formulations F1, F2, F3, and F4 showed the effect of homogenization time (min.) on encapsulation efficacy. D/P Ratio: Drug polymer ratio; R time: Retention time; AUC: Area under curve; %EE: Percentage entrapment efficiency; PVA: Polyvinyl alcohol

Formulation	D/P ratio	PVA%	Homogenization time(min.)	Centrifugation speed & time	R. time	AUC	% EE
Fa	1:1	1	2	40000(20)	2.15	137472	41.17
Fb	1:2	1	2	40000(20)	2.15	184975	55.60
Fc	1:3	1	2	40000(20)	2.15	165288	49.21
Fd	1:4	1	2	40000(20)	2.15	98222	27.42
A	1:2	1	2	40000(20)	2.1	14220	21
B	1:2	2	2	40000(20)	2.1	45735	59.46
C	1:2	3	2	40000(20)	2.1	23937	33.35
D	1:2	4	2	40000(20)	2.1	31181	42.03
F1	1:2	2	1	40000(20)	2.08	20347	34
F2	1:2	2	2	40000(20)	2.09	35370	52
F3	1:2	2	3	40000(20)	2.1	48566	67.68
F4	1:2	2	4	40000(20)	2.09	43234	61

Encapsulation efficiency and drug loading

**Figure 2:**
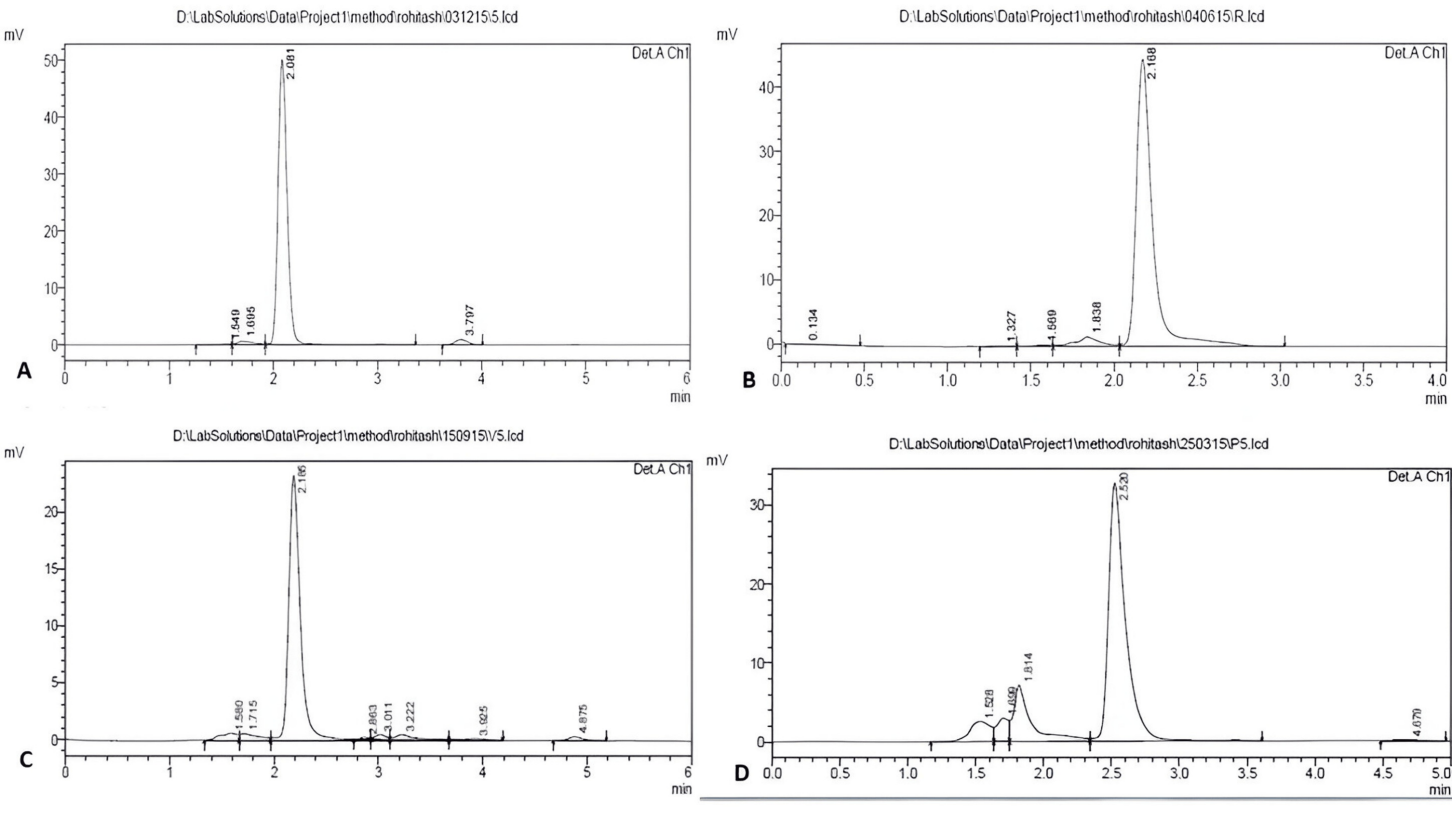
HPLC chromatogram of plain rifampicin (A) and nano formulation of rifampicin in mobile phase (B), vitreous fluid (C), and plasma (D) Graph A showed the retention time for plain rifampicin in the mobile phase (2.0 minutes), whereas graphs B, C, and D showed the retention time for rifampicin nanoformulation in the mobile phase (2.1 minutes), vitreous fluid (2.1 minutes), and plasma (2.5 minutes), respectively.

The maximum encapsulation efficiency obtained with the current set of experiments was 67.68% (Table 2) at 2% w/v concentration of PVA was selected for further characterization, and the drug loading was 42.28% w/w. The average particle size of the rifampicin formulation was 751.8 nm with an encapsulation efficiency of 68.71% w/w at 2% w/v concentration of PVA. The PDI of the developed nanoformulation showed 0.726. The Zeta potential of the rifampicin formulation was -12.18 mV with an encapsulation efficiency of 68.71% w/w at 2% w/v concentration of PVA. The encapsulation efficacy was estimated using HPLC methods. Figure 2 shows the HPLC chromatogram of plain rifampicin in the mobile phase and rifampicin nanoformulation in the mobile phase, vitreous fluid, and plasma, respectively. These are the representative chromatograms for all four metrics that show the retention time (RT) for rifampicin.

In-vivo assessment of intravitreal administration of free drug and nanoformulation

**Figure 3:**
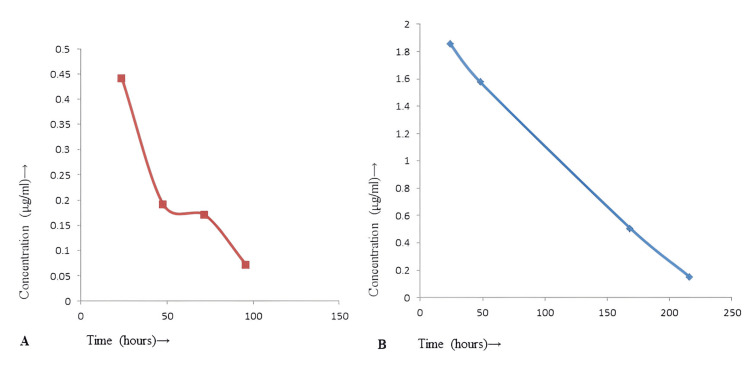
In-vivo release of plain rifampicin (A) and rifampicin nanoformulation (B) in the vitreous fluid after intravitreal administration Graph A showed the release of rifampicin (0.44 µg/ ml; 0.19µg/ ml; 0.17µg/ ml; 0.07µg/ ml at 24; 48; 72; 120 hours, respectively), whereas Graph B showed the release of rifampicin nanoformulation (1.86µg/ ml; 1.58µg/ ml; 0.51µg/ ml; 0.15µg/ ml at 24; 48; 168; 216 hours, respectively) in the vitreous after intravitreal administration in the rabbit eye.

The rifampicin-loaded nanoparticle demonstrated sustained release following intravitreal administration in the rabbit eye. The Cmax in the vitreous fluid for rifampicin nanoformulation was 1.86 µg/ml. We did not receive any significant plasma drug concentration following intravitreal administration of rifampicin and rifampicin nanoformulation. MIC of rifampicin nanoformulation (0.51 µg/ml) was received for seven days, whereas the free drug was for one day (Figure 3). The Cmax in vitreous fluid for free rifampicin was 0.44 µg/ml. A negligible drug concentration of free rifampicin was obtained after 24 hours in the vitreous.

## Discussion

Controlled drug delivery systems are one of the innovative areas of medical science [17]. It offers numerous advantages as compared to conventional dosage forms, including improved efficacy, reduced toxicity, improved patient compliance, and convenience. Such systems often use polymers as carriers for drugs. In recent years, polymeric nanoparticles have attained much importance as colloidal drug carriers owing to their smaller size, higher drug encapsulation efficiency, and low polymer consumption [18,19].

For the management of ocular tuberculosis, patients are administered first-line ATT routinely. There is no evidence to suggest whether systemic administration will lead to the attainment of adequate concentration in the vitreous. In this experimental proof-of-concept study, we have developed nanoparticles that would be sustained-release formulations, rifampicin-loaded PLGA nanoparticles intended for ocular tuberculosis. The developed nanoparticles of rifampicin were characterized for size, surface charge, entrapment efficiency, and drug loading.

Rifampicin-loaded nanoparticles were prepared by the single emulsion solvent evaporation method. The final formulation was developed after checking various experimental variables. We found that the @% PVA conc and 3 minutes of homogenization time provide the maximum rifampicin entrapment. Rifampicin-loaded nanoparticles with an average size of 786 nm and an encapsulation efficiency of 67.68% were selected for further studies. The nanoparticles exhibited low negative zeta potential (-12 mV), which could be due to the presence of terminal carboxylic groups of the PLGA. Zeta potential value is the measure of the surface charge of the particles, which ensures the stability of nanoparticles.

In this work, New Zealand White Rabbits were used for evaluating the sustained release of rifampicin nanoformulatin in the vitreous. The direct intravitreally administered drug was released in a sustained manner over 216 hours in the vitreous, and the drug did not reach the systemic circulation. The sustained action may be due to the prolonged biodegradation time of the polymer, PLGA [20]. The polymer-based nanoformulation acts as a depot for the gradual release of the drug [21]. Sustained availability of the formulation for seven days above the MIC holds a promise for direct administration at the site of action with infrequent administration.

Despite all the advantages of the proposed intravitreally administered nanoparticles, certain issues or limitations might need detailed study [22]. Further, it is associated with its specific complications. We made observations only for seven days. It needs to be seen if this sustained action can be achieved for longer periods and with other antitubercular drugs.

This proof-of-concept study has several limitations. Some of the major limitations of this study are the following:

Requires more experimental validation for sustained release nanoparticles of rifampicin. The two rabbits are not enough for the preclinical evaluation of PLGA nanoformulations of rifampicin. A combination of antitubercular drugs is required for the treatment of ocular tuberculosis. In this study, we have evaluated the sustained release of only rifampicin. Intravitreal administration of drugs is an invasive procedure that carries a risk of infection or which can potentially cause vision loss or even blindness.

## Conclusions

In conclusion, we have shown for the first time that rifampicin-loaded PLGA nanoparticles target specific sustained release into the vitreous for ocular tuberculosis. In this experimental proof-of-concept study, rifampicin-loaded PLGA nanoparticles showed intravitreal concentrations of rifampicin for up to seven days compared to a plain drug for one day in the vitreous of rabbit eyes. Whereas the plasma drug concentration was negligible at 24 hours after intravitreal administration of plain rifampicin and nanoformulation into rabbit eyes, revealing the target-specific action of rifampicin nanoformulation for ocular tuberculosis without systemic side effects.
